# Spontaneous speech and language measures as predictive biomarkers of clinically meaningful disease progression and neurodegeneration in Huntington’s disease

**DOI:** 10.1007/s00702-026-03143-x

**Published:** 2026-03-31

**Authors:** Arnau Puig-Davi, Carla Franch-Marti, Lara Caler-Gameiro, Jesus Perez-Perez, Gonzalo Olmedo-Saura, Jon Rodriguez-Antiguedad, Anna Vázquez-Oliver, Elisa Rivas-Asensio, Laura Perez-Carasol, Margarita Rubio-Romera, Yi Ji, Iñigo Ruiz-Barrio, Lidia Bojtos, Frederic Sampedro, Javier Pagonabarraga, Jaime Kulisevsky, Saul Martinez-Horta

**Affiliations:** 1 Institute of Neuroscience, Universitat Autònoma de Barcelona (UAB), Bellaterra, Spain; 2 Movement Disorders Unit, Neurology Department, Hospital de la Santa Creu i Sant Pau, Mas Casanovas 90, Barcelona, 08041 Spain; 3 Institut de Recerca Sant Pau (IR SANT PAU), Barcelona, Spain; 4 European Huntington’s Disease Network, Ulm, Germany; 5 Centro de Investigación en Red-Enfermedades Neurodegenerativas (CIBERNED), Barcelona, Spain; 6 Department of Medicine, Universitat Autònoma de Barcelona (UAB), Barcelona, Spain; 7 Neuroradiology Section, Radiology Department, Hospital Vall d’Hebron-IDI, Barcelona, Spain

**Keywords:** Huntington’s disease, Neurodegeneration, Biomarker, Speech, Cognitive impairment

## Abstract

Huntington’s disease (HD) is characterized by heterogeneous rates of clinical progression, complicating patient monitoring and clinical trial design. Although speech and language alterations are increasingly recognized as part of the HD cognitive phenotype, their value as short-term prognostic biomarkers of clinically meaningful disease progression and neurodegeneration remains unestablished. In this prospective 12-month longitudinal study, we investigated whether objectively quantified spontaneous speech and language measures predict short-term clinically meaningful progression and relate to biomarkers of neurodegeneration in HD. Eighty-six participants (42 manifest HD, 24 premanifest gene carriers, and 20 healthy controls) underwent baseline spontaneous speech assessment, structural MRI, and plasma neurofilament light chain (NfL) quantification. Clinically meaningful worsening was defined using validated minimal clinically important difference thresholds in the composite Unified Huntington’s Disease Rating Scale (cUHDRS). Spontaneous speech and language measures progressively deteriorated across disease stages and were associated with reduced cortico-subcortical gray matter volume in distributed associative and integrative regions. In manifest HD, logistic regression analyses revealed that baseline language integrity independently predicted clinically meaningful worsening at 12 months (OR = 3.840, 95% CI = 1.46–13.33; AUC = 0.783). Combining speech-derived measures with plasma NfL improved discrimination accuracy of individuals with accelerated clinical progression (AUC = 0.807). Spontaneous speech represents an early, accessible and sensitive marker of neurodegeneration in HD. The combination of speech and language derived measures and plasma NfL enables accurate identification of individuals at risk of accelerated, clinically meaningful disease progression, supporting their potential utility as short-term prognostic biomarkers for clinical trials enrichment and stratification.

**Supplementary Information:**

The online version contains supplementary material available at 10.1007/s00702-026-03143-x.

## Introduction

Huntington’s disease (HD) is a progressive, autosomal dominant neurodegenerative disorder caused by a CAG repeat expansion in the *HTT* gene (Ross and Tabrizi 2011). Basal ganglia atrophy is the main driver of fronto-striatal circuits disruption which leads to the progressive emergence of the characteristic motor, cognitive and neuropsychiatric symptoms of HD (Ross and Tabrizi 2011; Tabrizi et al. 2013; Paulsen et al. 2017; Baake et al. 2017). However, converging evidence from neuroimaging studies shows that neurodegeneration in HD also involves several cortical regions, which in turn also contributes to the clinical phenotype of HD (Wijeratne et al. 2021).

In line with the notion that HD is characterized by widespread neural dysfunction beyond the basal ganglia, several studies have highlighted a multidomain cognitive phenotype encompassing impairments in memory, visuospatial abilities, and language (Labuschagne et al. 2016; Martinez-Horta et al. 2020b, 2024; Puig-Davi et al. 2025). These deficits have been associated with heterogeneity in cognitive trajectories and profiles with potential implications for clinical trial design (Martinez-Horta et al. 2023, 2024). Within this multidomain profile, language impairment has historically received limited attention and was often regarded as a secondary consequence of motor speech or executive dysfunction (Podoll et al. 1988). Nonetheless, recent studies demonstrate that language alterations are frequent and multifaceted in HD, encompassing deficits in syntactic complexity, lexical access, semantic processing and sentence structure, some of which appear even in premanifest stages (Teichmann et al. 2005, 2008; Sambin et al. 2012; Hinzen et al. 2018; Gagnon et al. 2018; Giavazzi et al. 2018). These impairments typically worsen with disease progression and reflect the combined impact of linguistic, executive, and working memory disturbances (Teichmann et al. 2009; Jacquemot and Bachoud-Lévi 2021).

While earlier research linked these deficits primarily to basal ganglia dysfunction (Teichmann et al. 2005, 2006, 2008; Sambin et al. 2012; Hinzen et al. 2018), recent neuroimaging findings suggest that cortical degeneration, particularly in fronto-temporal and temporo-parietal areas, plays a critical role (Puig-Davi et al. 2025). This distributed pattern of cortical and subcortical involvement indicates that language alterations in HD reflect direct disruption of brain language networks (Jacquemot and Bachoud-Lévi 2021; Copland et al. 2021; Puig-Davi et al. 2025).

Despite increasing recognition of these deficits, the prognostic value of linguistic assessment as a biomarker of neurodegeneration and clinical progression in HD remains poorly understood. Previous studies have all been cross-sectional, focused on isolated linguistic domains, and have rarely examined the relationship between linguistic variables and biomarkers of neurodegeneration, such as atrophy measured by brain MRI or plasma neurofilament light chain (NfL) levels.

The present study aims to: (1) Study the relationship of spontaneous speech and language measures with neurodegeneration biomarkers across disease stages; and (2) evaluate whether specific spontaneous speech and language features can be used as short-term disease prognostic markers.

## Methods

### Participants

The study sample comprised 66 HD gene expansion carriers (HDGEC), including 42 individuals with manifest HD (mHD) and 24 premanifest carriers (preHD), together with 20 healthy control participants (HC). All participants were recruited from the HD Clinic of the Movement Disorders Unit at Hospital de la Santa Creu i Sant Pau. Eligible participants were required to carry a fully penetrant HTT gene expansion (> 39 CAG repeats) and to have no history of neurological conditions other than HD. Additional exclusion criteria included previous traumatic brain injury, neurosurgical procedures, epilepsy, substance abuse, or uncontrolled systemic medical illness.

### Assessments

Clinical and sociodemographic information, including age, sex and years of education was collected for all participants. Motor symptom severity was evaluated using the Unified Huntington’s Disease Rating Scale – Total Motor Score (UHDRS-TMS) (Huntington Study Group 1996), and functional status with the Total Functional Capacity (TFC) scale (Shoulson and Fahn 1979). Participants were classified into disease stages 0 to 3 according to the biological framework of the Huntington’s Disease Integrated Staging System (HD-ISS) (Tabrizi et al. 2022).

Disease burden was estimated using the CAG–age product (CAP) score, calculated as age x (CAG-33.66) (Zhang et al. 2011). Cognitive performance was assessed using the Symbol Digit Modalities Test (SDMT) and Stroop Word Reading Test (SWRT) as measures of processing speed and executive functioning (Landwehrmeyer et al. 2017), as well as to calculate the composite UHDRS score (cUHDRS) (Schobel et al. 2017). All the assessments comprising the cUHDRS (UHDRS-TMS, TFC, SDMT and SWRT) were also collected one year after inclusion in 37 participants in HD-ISS 2 and 3 in order to calculate the minimal clinically important difference (MCID) in the cUHDRS at 12 months of follow-up (Hamilton et al. 2023). The thresholds used to estimate MCID were calculated using anchor-based methods in a prospective, well-characterized HD sample. At the individual level, a 0.64-point decline in the cUHDRS for HD-ISS 2 and a 0.94-point decline for HD-ISS 3 at 12 months is considered a clinically meaningful change (Hamilton et al. 2023).

Global cognition was evaluated with the Parkinson’s Disease–Cognitive Rating Scale (PD-CRS), a brief cognitive screening instrument originally developed for Parkinson’s disease and subsequently validated in Huntington’s disease. It provides a total score (range 0-134), with higher scores indicating better performance. The scale comprises two main subscores: a fronto-subcortical subscore, reflecting executive and attentional functions typically affected in HD, and a posterior cortical subscore, reflecting language and visuospatial abilities (Pagonabarraga et al. 2008; Martinez-Horta et al. 2020a).

Spontaneous speech and language were assessed using the Cookie Theft picture-description subtest of the Boston Diagnostic Aphasia Examination, 3rd ed. (Goodglass et al. 2001). Participants were instructed to describe the picture freely in Spanish for 60 s. Descriptions were audio-recorded and orthographically transcribed verbatim by a trained speech therapist. Transcripts were corrected according to the BDAE guidelines. Additionally, transcripts were subjected to the exhaustive multi‐index framework proposed by Hinzen et al. (2018), which evaluates four linguistic domains: fluency, reference, connectivity and concordance. The fluency domain reflects disturbances in the flow of speech, including pauses, repetitions and word truncations. The reference domain captures the appropriate use of language to identify characters and objects and to establish discourse topics. The connectivity domain reflects how clauses are grammatically combined within and across sentences. The concordance domain indexes grammatical agreement and morphosyntactic integrity. A Spontaneous Language Composite Score (SLCS) was computed by averaging the normalized z-scores from the four linguistic domains. Two measures were obtained as measures of speech: Words per minute (WPM) as a measure of speech rate and mean utterance length in words (MLU-w). All transcripts were anonymized and scored by two independent investigators. Inter-rater reliability was excellent (Cohen’s κ > 0.90); any discrepancies were resolved by consensus (Detailed information can be found in Supplementary Material 1). The combined time required for orthographic transcription and application of the linguistic scoring framework was approximately 15 min per participant when performed by a trained rater.

### Neuroimaging acquisition and preprocessing

T1-weighted structural brain images were collected using a 3.0 Tesla Philips Ingenia magnetic resonance system. Data acquisition employed a MPRAGE sequence (TR/TE of 12.65/7.08 milliseconds, flip-angle of 8º, field of view of 23 cm, acquisition matrix of 256 × 256 and a slice thickness of 1 mm). Morphometric analyses based on voxel-wise comparisons (VBM) were performed using SPM12 (Statistical Parametric Mapping, http://www.fil.ion.ucl.ac.uk/spm). From the acquired T1-weighted datasets, gray matter volume (GMV) probability maps were computed and subsequently normalized into standard Montreal Neurological Institute space through high-dimensional registration implemented in the DARTEL framework. Finally, to mitigate the impact of anatomical variability across participants, the resulting GMV maps were convolved with an isotropic Gaussian smoothing kernel of 8 mm full width at half maximum.

### Biosamples collection and processing

Blood samples were collected on-site into EDTA tubes and centrifuged at 2000 g for 10 min. Plasma was aliquoted into polypropylene tubes, and stored at − 80 °C until analysis, following international consensus guidelines. Plasma neurofilament light chain (NfL) concentrations were quantified using the Simoa Human NF-light Advantage kit (Catalogue No. 104073) on the SR-X platform (Single Molecule Array; Quanterix, Lexington, MA, USA), in accordance with the manufacturer’s protocol. All the samples were analyzed in duplicate, and the intra-assay coefficient of variation was below 15%.

### Statistical analysis

Descriptive and clinical variables were analyzed using non-parametric statistics. Group comparisons for continuous variables were performed using the Kruskal-Wallis test with Conover-Iman post hoc comparisons, while categorical variables were analyzed using χ^2^. Data are reported as median values with interquartile ranges (IQR). Plasma NfL concentrations were log-transformed prior to analysis and adjusted for body mass index.

Multivariate linear regression models were used to examine the predictive value of cross-sectional and linguistic measures to cognitive decline and neurodegeneration measures. All predictors were standardized to z-scores to facilitate the interpretability and comparability of the model coefficients. Model assumptions, including linearity, normality and homoscedasticity of residuals, and absence of multicollinearity were checked and met. Model selection was guided by the Akaike Information Criterion (AIC), with preference given to the model yielding the lowest AIC value. Statistical significance was defined at α = 0.05, with p-values corrected for multiple comparisons using the Benjamini-Hochberg false discovery rate (FDR) method.

Binary logistic regression models were used to assess the predictive value of cross-sectional variables for significant clinical worsening according to longitudinal MCID in the cUHDRS at 12 months (Hamilton et al. 2023). Model assumptions, including linearity of continuous predictors with the logit of the outcome and absence of multicollinearity among covariates, were verified and met. All predictor variables were standardized to z-scores to facilitate the interpretability and comparability of the model coefficients. Predictive performance of the final models was evaluated using receiver operating characteristic (ROC) curve analysis. The area under the curve (AUC) was reported with 95% confidence intervals estimated by stratified non-parametric bootstrap resampling with 2,000 iterations. Sensitivity, specificity, positive predictive value (PPV), and negative predictive value (NPV) were calculated at the optimal cut-off determined by the Youden index. P-values were corrected for multiple comparisons using the Benjamini-Hochberg FDR method.

Voxel-wise GMV measures derived from VBM analyses were introduced into general linear models (GLM) to investigate structural brain correlates of the linguistic indices. These models included Total Intracranial Volume, CAP score, years of education and sex as covariates of no interest. Statistical significance was set at *p* < 0.05 after family-wise error (FWE) correction at the cluster level using random field theory, as implemented in SPM12.

All analyses were performed in R software version 4.2.2 (R Project for Statistical Computing).

## Results

### Sample characteristics

Clinical and sociodemographic characteristics of the different groups are presented in Table 1. All the participants were bilinguals (Spanish-Catalan) with a native level for Spanish. There was a consistent Spanish-dominant profile across all groups, with an average of 77.9% (χ2(4,86) = 0.52, *p* = 0.972).

**Table 1:** Clinical and sociodemographic characteristics

	Healthy controls *N* = 20	HD-ISS 0 *N* = 14	HD-ISS 1 *N* = 10	HD-ISS 2 *N* = 11	HD-ISS 3 *N* = 31	*p*-value
Age	44 (40, 59)^a^	33 (30, 39)^f, g^	44 (37, 53)	48 (42, 54)	57 (46, 62)	< 0.001
Sex						
Male	9 (45%)	2 (14%)	5 (50%)	6 (55%)	14 (45%)	0.210
Female	11 (55%)	12 (86%)	5 (50%)	5 (45%)	17 (55%)	
Education (years)	14.5 (11.0, 18.0)	18.0 (14.0, 18.0)^f, g^	17.5 (16.0, 18.0)^i^	14.0 (13.0, 16.0)	14.0 (10.0, 17.0)	0.013
Handedness						
Right	19 (95%)	14 (100%)	9 (90%)	11 (100%)	28 (90%)	0.7
Left	1 (5.0%)	0 (0%)	1 (10%)	0 (0%)	3 (9.7%)	
CAG	NA	42.00 (40.00, 43.00)	42.00 (41.00, 42.00)	43.00 (41.00, 43.00)	42.00 (42.00, 46.00)	0.279
CAP Score	NA	293 (275, 300)^e, f,g^	364 (336, 448)^i^	420 (396, 458)^j^	509 (470, 576)	< 0.001
UHDRS-TMS baseline	0 (0, 0)b, c,d	0 (0, 0)^e, f,g^	1 (0, 4)^h, i^	11 (7, 19)^j^	46 (30, 59)	< 0.001
UHDRS-TMS 12 months	-	-	-	17 (11, 24)	44 (35, 60)	NA
TFC baseline	13.00 (13.00, 13.00)d	13.00 (13.00, 13.00)g	13.00 (13.00, 13.00)i	13.00 (13.00, 13.00)j	10.00 (7.00, 11.00)	< 0.001
TFC 12 months	-	-	-	13.00 (13.00, 13.00)	9.00 (7.00, 11.00)	NA
PD-CRS	108 (98, 117)^c, d^	112 (98, 119)f, g	115 (106, 120)h, i	83 (70, 96)^j^	73 (65, 83)	< 0.001
SDMT baseline	56 (47, 65)^c, d^	55 (47, 66)^f, g^	55 (52, 56)h, i	32 (31, 40)^j^	22 (13, 26)	< 0.001
SDMT 12 months	-	-	-	35 (30, 43)	20 (11, 31)	NA
cUHDRS baseline	18.2 (16.6, 19.8)^c, d^	18.0 (17.1, 18.6)^f, g^	17.9 (17.4, 18.4)^h, i^	13.6 (12.3, 14.4)^j^	7.8 (4.3, 9.9)	< 0.001
cUHDRS 12 months	-	-	-	13.3 (12.3, 13.9)	6.7 (3.8, 10.7)	NA
MCID in cUHDRS at 12 months						
Yes	-	-	-	2 (22.2%)	14 (50%)	NA
No	-	-	-	7 (77.8%)	14 (50%)	
NfL (pg/ml)	4 (3, 6)^b, c,d^	7 (4, 9)^e, f,g^	15 (9, 26)^i^	17 (14, 23)^j^	26 (19, 28)	< 0.001
Words per minute	143 (125, 160)^c, d^	153 (128, 163)^f, g^	145 (137, 153)^h, i^	97 (89, 127)	92 (65, 114)	< 0.001
Mean length utterance-words	10.56 (9.50, 12.59)^d^	11.92 (11.70, 14.18)^f, g^	13.32 (8.58, 15.30)^h, i^	8.54 (7.43, 11.22)	8.62 (7.14, 11.50)	< 0.001
Boston Complexity Index	1.48 (1.30, 1.79)^c, d^	1.77 (1.45, 1.92)^f, g^	1.53 (1.31, 1.77)^h, i^	1.14 (1.00, 1.28)	1.13 (0.90, 1.38)	< 0.001
Spontaneous Language Composite Score	-0.32 (-0.53, -0.12)^c, d^	-0.61 (-0.76, -0.25)^e, f,g^	-0.14 (-0.64, 0.06)^h, i^	0.13 (-0.14, 0.57)	0.40 (-0.10, 1.17)	< 0.001

Data expressed as median (interquartile range) for continuous variables and frequency (%) for categorical variables. The Benjamini-Hochberg method was applied for multiple comparison correction, yielding adjusted p-values < 0.05

*UHDRS-TMS* Unified Huntington’s Disease Rating Scale – Total Motor Score; *TFC* total functional capacity; *PD-CRS* Parkinson’s Disease Cognitive Rating Scale; *SDMT* symbol digit modalities test; *cUHDRS* composite UHDRS; *MCID* minimal clinically important difference; *NfL* neurofilament light chain

^a^Controls vs. HD-ISS 0 *p <* 0.05, ^b^Controls vs. HD-ISS 1 *p <* 0.05, ^c^Controls vs. HD-ISS 2 *p <* 0.05, ^d^Controls vs. HD-ISS 3 *p <* 0.05, ^e^HD-ISS 0 vs. HD-ISS 1 *p <* 0.05, ^f^HD-ISS 0 vs. HD-ISS 2 *p <* 0.05, ^g^HD-ISS 0 vs. HD-ISS 3 *p <* 0.05, ^h^HD-ISS 1 vs. HD-ISS 2 *p <* 0.05, ^i^HD-ISS 1 vs. HD-ISS 3 *p <* 0.05, ^j^HD-ISS 2 vs. HD-ISS 3 *p <* 0.05

**Fig. 1:**
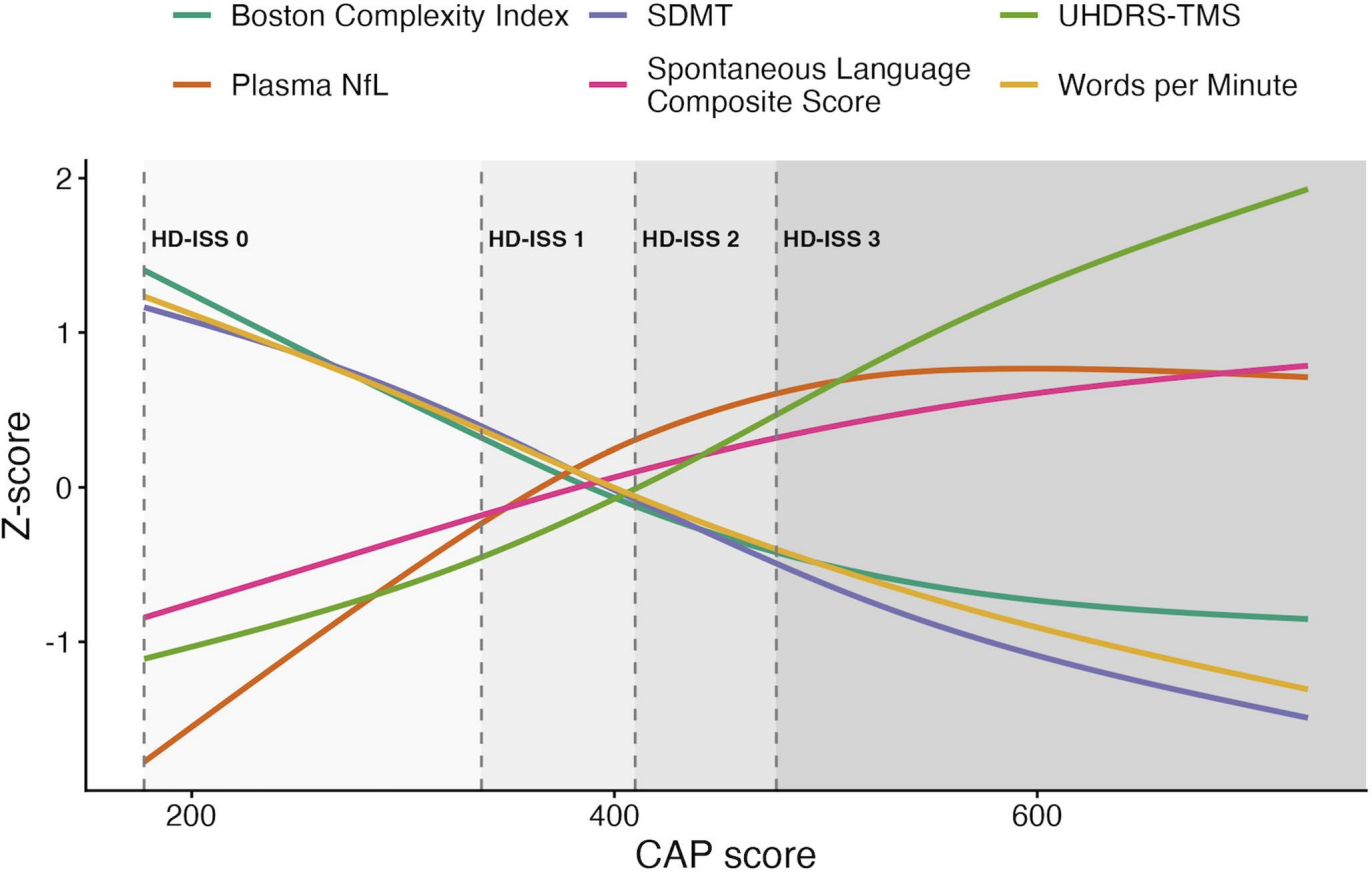
Cross-sectional associations between clinical measures, plasma NfL levels and disease severity across HD-ISS stages. Smoothed curves represent model-based estimates derived from cross-sectional data and are shown for illustrative purposes; all statistical inferences were based on linear models

Significant group differences were observed across all clinical domains. Overall, HD-ISS 2–3 showed significantly worse scores in cognitive, linguistic and motor variables than HD-ISS 0–1 groups and HC. Focusing on speech and linguistic scores, both speech rate (WPM) and MLU-w were reduced in HD-ISS 2–3 relative to HC and HD-ISS 0–1, while Boston complexity index showed a further decline from stage 2 to stage 3. Differences in SLCS were already detectable between HD-ISS stage 0 and 1. These differences became more pronounced in stages 2 and 3, with both showing a progressive decline compared to stage 0 and HC. This decline was mainly driven by increased errors in fluency and reference domains in stages 2 and 3, reduced grammatical complexity in stages 2 and 3 compared to earlier stages, and increased errors in the concordance domain in stage 3 compared to all other groups (Supplementary Table 1). Figure 1 illustrates the estimated trajectory of different variables across HD-ISS stages.

### Cross-sectional associations in symptomatic stages (HD-ISS 2–3)

Because differences relative to controls were evident from the symptomatic stages onward, and because clinically meaningful change in cUHDRS has been validated only in this population, to avoid potential confounding effects from combining presymptomatic and symptomatic participants in linear models, all subsequent analyses were restricted to individuals in disease stages 2 and 3.

Multivariate linear regression models, adjusting for UHDRS-TMS, CAP score and years of education identified several linguistic components as independent predictors of cognitive performance according to the PD-CRS. SLCS showed the strongest association with PD-CRS total (β = -0.334, 95% CI = -0.574 – − 0.094, *p* = 0.009) and posterior cortical scores (β = -0.677, 95% CI = -1.050 – − 0.303, *p* = 0.002). WPM (β = 0.317, 95% CI = 0.131–0.504, *p* = 0.002) and MLU-w (β = 0.202, 95% CI = 0.039–0.365, *p* = 0.016) showed a similar pattern with PD-CRS total score but were not associated with PD-CRS posterior cortical score.

### Prediction of longitudinal significant clinical change

**Fig. 2:**
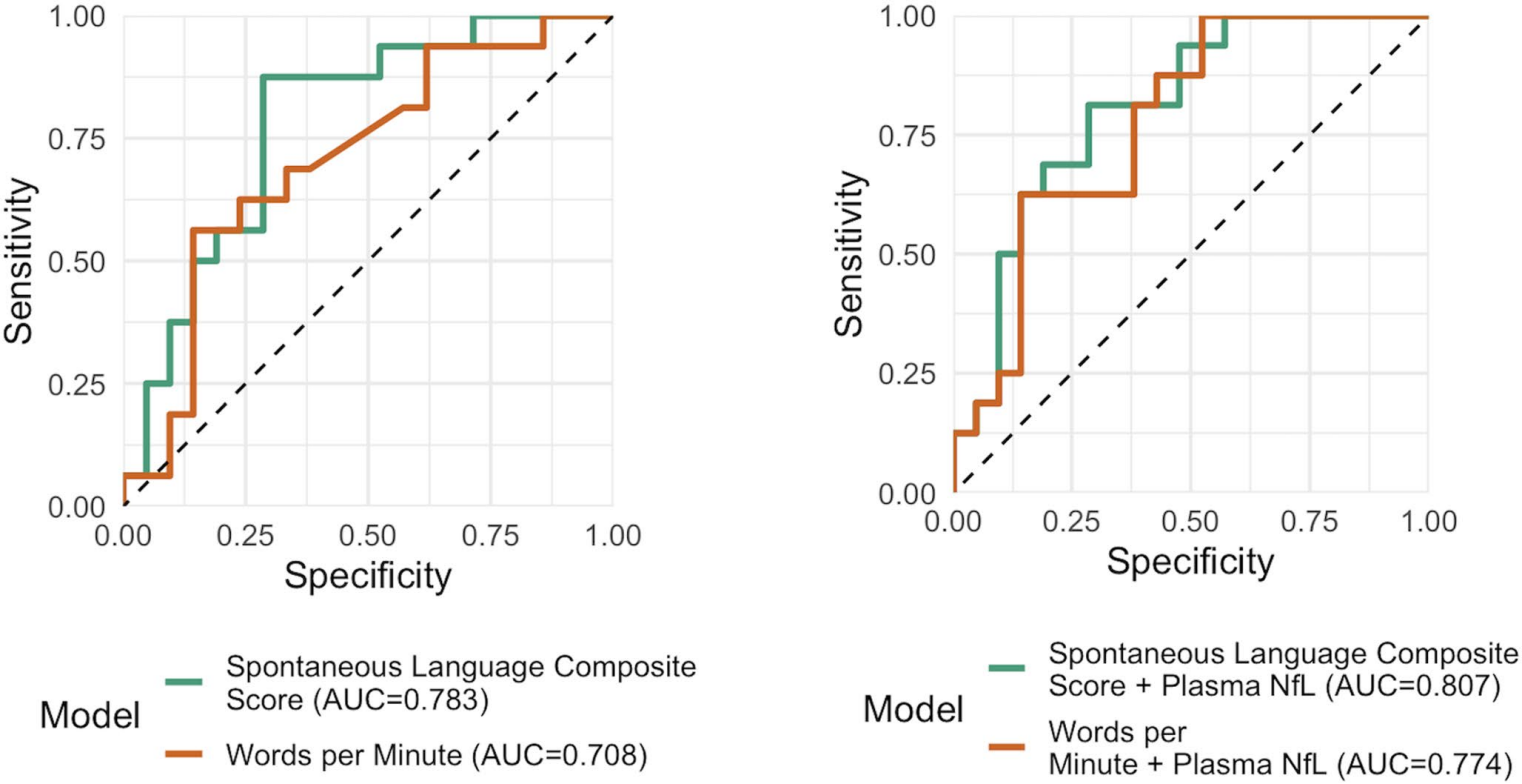
ROC curves representation of the performance of the different binary logistic regression models to predict significant clinical change in the cUHDRS at 12 months

Binary logistic regression models were then built to predict significant clinical worsening according to the change in the cUHDRS in HD-ISS stages 2–3 at 12 months. Of the 37 individuals with available follow-up data, 16 (43.2%) met criteria for significant clinical worsening (detailed results in Table 1). A first model with CAP score and years of education as covariates was built to confirm that SLCS (OR = 4.91, 95% CI = 1.47–16.33, *p* = 0.030) and WPM (OR = 0.305, 95% CI = 0.107–0.875, *p* = 0.036) were independent predictors of significant clinical worsening. Once confirmed, logistic models were re-analyzed with SCLS and WPM independently. Worse scores in baseline SLCS and WPM increased the risk of significant clinical worsening according to the cUHDRS at 12 months (SLCS OR = 3.840, 95% CI = 1.46–13.33, *p* = 0.030; WPM OR = 0.410, 95% CI = 0.150–0.910, *p* = 0.046). ROC curves, AUC scores, PPV and NPV were used to evaluate performance of the two models. Adding baseline NfL plasma levels improved the accuracy of the models as shown in Table 2; Fig. 2.

**Table 2:** Binary logistic regression model ability to predict significant clinical change in the cUHDRS at 12 months

	AUC (CI 95%, bootstrap)	Threshold	Sensitivity	Specificity	PPV	NPV
Spontaneous language composite score	0.783 (0.622–0.911)	0.358	0.875	0.714	0.700	0.882
Words per minute	0.708 (0.521–0.869)	0.516	0.562	0.857	0.750	0.720
Spontaneous language composite score + NfL	0.807 (0.646–0.932)	0.380	0.812	0.714	0.684	0.833
Word per minute + NfL	0.774 (0.610–0.914)	0.524	0.625	0.857	0.769	0.750

AUC confidence intervals were derived using stratified non-parametric bootstrap resampling with 2,000 iterations. The optimal threshold was determined using the Youden index

*AUC* area under the curve; *CI* confidence interval; *PPV* positive predictive value; *NPV* negative predictive value; *NfL* neurofilament light chain

### Association with biomarkers of neurodegeneration

**Fig. 3:**
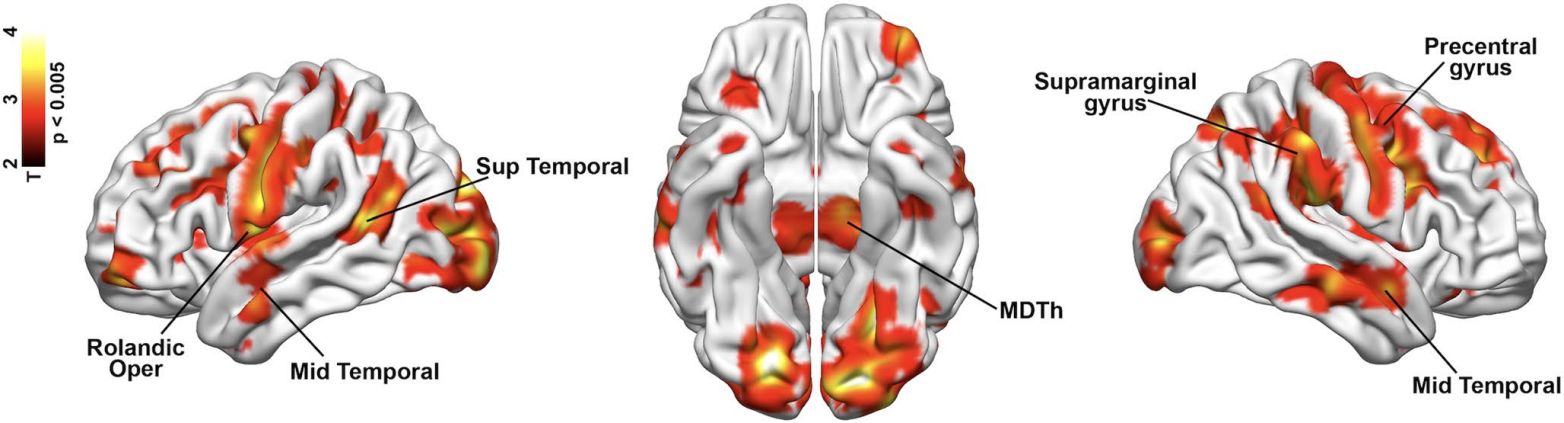
Gray matter volume correlates of spontaneous language compostie score (FWE corrected; *p* < 0.05). *MDTh* medial dorsal nucleus of the thalamus

Voxel-wise VBM analysis revealed significant associations between worse scores in the SLCS and reduced GMV in bilateral medial dorsal nucleus of the thalamus, bilateral mid temporal gyrus, bilateral precentral gyrus, left superior temporal lobe, left inferior frontal gyrus and right supramarginal gyrus (Fig. 3). SLCS scores were not significantly associated with Plasma NfL levels (β = 0.03, 95% CI = -0.58–0.63, *p* = 0.932).

In addition to these cross-sectional biomarker associations, we evaluated whether baseline regional GMV and plasma NfL levels predicted longitudinal clinically meaningful worsening. Binary logistic regression models adjusting for age, years of education and CAP score, revealed that preservation of GMV in the bilateral medial dorsal nucleus of the thalamus was associated with a lower risk of significant clinical worsening in the cUHDRS at 12 months (OR = 0.24, 95% CI = 0.07–0.841, *p* = 0.025). Preservation of GMV in right supramarginal gyrus was also associated with a lower risk of clinical worsening (OR = 0.20, 95% CI = 0.05–0.75, *p* = 0.017). Plasma NfL was not able to predict clinically significant change (OR = 4.14, 95% CI = 0.78–21.94, *p* = 0.095).

## Discussion

This longitudinal study demonstrates that spontaneous speech-derived measures are sensitive markers of short-term clinical prognosis and neurodegeneration in HD. Specifically, lower SLCS scores were independently associated with poorer global cognitive status and, in particular, with greater decline in posterior cortical functions compared to other classical measures of HD progression, such as WPM. Moreover, SLCS was further linked to a more widespread pattern of structural brain compromise and its combination with plasma NfL levels was able to accurately predict clinically meaningful worsening on the cUHDRS at 12 months. WPM also predicted short-term clinically meaningful change in the cUHDRS, though with lower accuracy.

Importantly, the nature of the speech task used in this study (a description of a predetermined picture) provides a controlled communicative context that constrains the range of possible content, reducing the cognitive demand associated with discourse planning and topic generation. This structured format arguably constitutes a relatively “easier” task compared to truly free-form spontaneous speech.

The current literature on speech and language in HD has relied on cross-sectional approaches, primarily aimed at characterizing the linguistic and motor-speech alterations that emerge from the earliest stages of the disease. In-depth analyses have shown that HD leads to a progressive disintegration of linguistic structure and simplification of narrative discourse, including reduced syntactic variety, lexical-semantic deficits and discourse-level disruptions (Hinzen et al. 2018; Gagnon et al. 2018; Puig-Davi et al. 2025). In parallel, speech biomarker studies have reported that acoustic measures are sensitive to disease stage, capturing subtle motor-speech and prosodic changes that are lineal to clinical progression (Kouba et al. 2023). Only one small longitudinal study has documented a change in speech performance in preHD over time (Saft et al. 2024), but its predictive capability has never been assessed.

The association between the SLCS and the bilaterally distributed pattern of structural brain involvement at the GMV level suggests that the cognitive processes underlying spontaneous speech generation rely on networks that extend beyond classical language areas. The link between SLCS and atrophy in the mediodorsal thalamic nucleus and right supramarginal gyrus, together with the relationship of these three variables to short-term clinical progression, may indicate that the compromise of these structures plays a key role in the apparent acceleration of clinical decline observed in the subgroup of mHD individuals who tend to progress more rapidly (Martinez-Horta et al. 2020b).

Our findings that lower SLCS relates to both short-term clinical worsening and more widespread neurodegeneration in HD align with the growing view that clinical and cognitive decline in HD arises from disruptions of distributed cortico-subcortical networks beyond the striatum. The mediodorsal thalamus is a key prefrontally connected hub supporting executive control and integration, and volume loss in this region is detectable in early manifest HD and predicts greater neurocognitive and motor dysfunction (Furlong et al. 2020). Likewise, FDG-PET studies have reported hypometabolism in parietal and thalamic regions as patients approach clinical onset (Feigin et al. 2007). Thus, the association between SLCS and mediodorsal thalamic atrophy is biologically plausible, as degeneration in this region likely accelerates clinical progression. Similarly, the link between SLCS and right supramarginal gyrus atrophy situates the SLCS within posterior association networks that have been associated with greater cognitive impairment (Martinez-Horta et al. 2020b). Notably, posterior cortical atrophy in HD can occur somewhat independently of CAG-repeat length and age, implying that factors beyond the primary genetic burden may contribute to the accelerated decline observed in certain individuals (Martinez-Horta et al. 2020b). Functionally, the right supramarginal gyrus is involved in attentional control, visuospatial processing, and language integration (especially prosody and contextual understanding), reflecting its role within a broad cognitive network (Krall et al. 2015). Damage to the supramarginal gyrus and adjacent inferior parietal cortex could therefore undermine not only language fluency but also other posterior cortical functions. Consistent with this, longitudinal analyses suggest that cortical atrophy is a stronger predictor of cognitive decline in HD than striatal loss alone (Johnson et al. 2021). For instance, patients with comparable striatal degeneration show more rapid cognitive decline when greater atrophy is present in parietal and occipital cortices. It is important to note that other factors such as genetic modifiers, somatic CAG repeat instability, cognitive reserve and bilingual experience could also modulate both structural vulnerability and compensatory mechanisms within cognitive networks (Migliore et al. 2022; Donaldson et al. 2026). Although years of education were included as a covariate in our models, more comprehensive measures of cognitive reserve and sociolinguistic background were not formally assessed. Therefore, replication in independent cohorts, particularly those with different linguistic and cultural contexts, will be essential to determine the generalizability and robustness of these neuroanatomical associations.

Plasma NfL, a general marker of neurodegeneration, increases as HD pathology extends beyond the striatum and is strongly related to symptom progression in preHD and early HD (Parkin et al. 2024). Interestingly, combining a marker of clinical heterogeneity (SLCS) with an established marker of neurodegeneration (NfL) improved the prediction of 12-month clinically significant decline in the cUHDRS. Spontaneous speech measures independently predicted near-term progression and their combination with NfL improved the AUC above 0.8, which is considered excellent. This performance must be interpreted in light of the very short prediction window. Anticipating clinically meaningful change within just 12 months represents a stringent challenge, and in this narrow timeframe, speech-based measures outperformed plasma NfL levels when analyzed independently. Although NfL is a robust biomarker in preHD, its predictive capacity diminishes in symptomatic stages due to plateau effects, limiting its ability to capture short-term clinical worsening on its own (Parkin et al. 2021; Li et al. 2023). This attenuation may reflect not only a ceiling effect, but also the limited topographical and functional specificity of NfL with respect to the cognitive domains involved. If SLCS-related processes are relatively independent of CAG repeat length and global disease burden, they may be indexing a component of cognitive heterogeneity in HD that is possibly mediated by additional pathological mechanisms such as Tau pathology (Martinez-Horta et al. 2023, 2024). These findings position language-derived metrics as complementary yet distinct within the biomarker landscape, with the advantage of detecting clinically relevant decline over windows where other markers fall short. Together, these results suggest that spontaneous speech captures vulnerability across thalamo-prefrontal and inferior parietal circuits, providing a sensitive and clinically meaningful prognostic tool in HD.

Spontaneous language assessment is simple, non-invasive and scalable that may support monitoring of disease progression and identification of patients with accelerated clinical decline which could have several implications for clinical trial design. On one hand, it could be highly valuable for stratifying participants and assessing the effects of experimental treatments according to their progression profiles (Hamilton et al. 2023; Roiboit and Stout 2025). On the other hand, it could help advance our understanding of the biological mechanisms underlying clinical heterogeneity, ultimately guiding the development of more personalized therapeutic approaches. Finally, if patients who follow the expected disease course are analyzed together with those showing an accelerated progression pattern, it may become more difficult to detect true treatment effects at the group level. Because treatments likely exert differential responses across these subgroups, treating all participants as if they follow the same trajectory can dilute true therapeutic effects in group-level analyses.

Several limitations should be acknowledged. Firstly, although the sample was well-characterized and prospectively followed, the 12-month interval may be relatively short to fully capture long-term trajectories of disease progression. Secondly, although cross-sectional differences in language measures were observed across disease stages, the longitudinal prognostic analyses were restricted to individuals in HD-ISS stages 2 and 3. Therefore, evidence supporting the use of spontaneous speech and language measures as prognostic biomarkers in premanifest stages (HD-ISS 0–1) remains limited. Future longitudinal studies specifically targeting earlier stages are needed to determine whether these metrics may have predictive value prior to manifest disease. Importantly, disease progression in HD is known to be heterogeneous partly due to factors such as somatic CAG instability and genetic modifiers of disease onset and progression. These sources of biological variability were not explicitly modeled in the present study and may contribute to differential rates of clinical and cognitive decline, potentially influencing the observed associations. The linguistic framework, while comprehensive, was applied to a single speech elicitation task, which may limit generalizability to other communicative contexts. Neuroimaging analyses were cross-sectional, restricting causal inferences about the temporal relationship between speech changes and structural decline. Similarly, although plasma NfL provided an independent molecular anchor, additional biomarkers (e.g., functional connectivity or other fluid markers) could enhance interpretability. Finally, the cohort size, while comparable to previous studies, limits statistical power for subgroup analyses and may restrict the detection of subtle effects. Future research should replicate these findings in larger multicenter cohorts, extend the follow-up period and incorporate diverse speech tasks. Automated pipelines using natural language processing and speech recognition will be critical to enable scalable deployment. Such approaches would facilitate their integration into longitudinal monitoring frameworks and clinical trials, supporting the use of language-based metrics as digital biomarkers sensitive to cognitive and neurobiological changes across disease stages.

In conclusion, spontaneous speech emerges as an independent, sensitive marker of short-term clinical prognosis and neurodegeneration in HD. Incorporating linguistic assessments offers a potentially accessible and cost-effective approach to further understand clinical heterogeneity and for the enrichment of clinical trials design

## Supplementary Information

Below is the link to the electronic supplementary material.

Supplementary Material 1

## Data Availability

The data that support the findings of this study are available on request from the corresponding author. The data are not publicly available due to ethical restrictions.
