# Supplementary material for: Spontaneous speech and language measures as predictive biomarkers of clinically meaningful disease progression and neurodegeneration in Huntington’s disease
Source: J Neural Transm (Vienna). 2026 Mar 31;133(7):1519–29. doi: 10.1007/s00702-026-03143-x (PMC13428781; doi:10.1007/s00702-026-03143-x)
Supplement: Supplementary file 1 — Supplementary Material 1 [file 702_2026_3143_MOESM1_ESM.docx]

**Article Title: Spontaneous Speech and Language Measures as Predictive Biomarkers of Clinically Meaningful Disease Progression and Neurodegeneration in Huntington’s Disease**

**Journal Name: Journal of Neural Transmission**

Arnau Puig-Davi^1,2,3,4,5†^; Carla Franch-Marti^2,3,4†^; Lara Caler-Gameiro^2^; Jesus Perez-Perez^1,2,3,4,5^; Gonzalo Olmedo-Saura^2,3,4,5,6^; Jon Rodriguez-Antiguedad^2,3,5^; Anna Vázquez-Oliver^2,3,5^; Elisa Rivas-Asensio^2,3,4,5^; Laura Perez-Carasol^2,3,4,5,6^; Margarita Rubio-Romera^2,3,4^; Yi Ji^2,3,4^; Iñigo Ruiz-Barrio^2,3,4,5^; Lidia Bojtos^2,3,4,5,6^; Frederic Sampedro^2,3,5^; Javier Pagonabarraga^2,3,4,5,6^ Jaime Kulisevsky^1,2,3,4,5,6*^; Saul Martinez-Horta^1,2,3,4,5,6*^

1. Institute of Neuroscience, Universitat Autònoma de Barcelona (UAB), Bellaterra, Spain

2. Movement Disorders Unit, Neurology Department, Hospital de la Santa Creu i Sant Pau, Barcelona, Spain

3. Institut de Recerca Sant Pau (IR SANT PAU), Barcelona, Spain

4. European Huntington’s Disease Network, Ulm, Germany

5. Centro de Investigación en Red-Enfermedades Neurodegenerativas (CIBERNED), Spain

6. Department of Medicine, Universitat Autònoma de Barcelona (UAB), Barcelona, Spain

†These authors contributed equally to this work

***Corresponding Authors:** Jaime Kulisevsky and Saul Martinez-Horta

E-mail: [Jaime.Kulisevsky@uab.cat](mailto:Jaime.Kulisevsky@uab.cat) and [smartinezho@santpau.cat](mailto:smartinezho@santpau.cat)

**SUPPLEMENTARY MATERIAL**

**INDEX**

1. **Supplementary Material 1 –** Detailed information of the picture-description task and scoring system of the different speech and language derived measures
2. **Supplementary Table 1 –** Domain scores of the Spontaneous Language Composite Score across groups

**Supplementary Material 1 –** Detailed information of the picture-description task and scoring system of the different speech and language derived measures

Spontaneous speech and language were assessed with the Cookie Theft picture‐description subtest of the Boston Diagnostic Aphasia Examination, 3rd ed. Each participant had 60 s to describe the scene spontaneously in Spanish. Descriptions were recorded on head-mounted microphone (44.1 kHz, 16-bit) and orthographically transcribed verbatim by a trained speech therapist. Transcripts were segmented into utterances with a slash “/” marking each pause-terminated prosodic unit, per the BDAE guidelines. For each utterance we coded: v: empty utterances conveying no picture-relevant information, sc: sub-clausal utterances lacking a full Subject–Verb frame but containing content words, cl: single-clause utterances with one finite verb, mcl: multi-clause utterances with > 1 finite verb, agr: agrammatical omissions of obligatory grammatical morphemes or function words. A Complexity Index was computed as total clauses divided by (total utterances – v). Words per minute (WPM) was recorded as a measure of speech rate. Mean utterance length in words (MLU-w) and was also recorded. Additionally, transcripts were subjected to the exhaustive multi‐index framework proposed by Hinzen et al. (2018), which evaluates four linguistic domains. The fluency domain captured prolongations, filled pauses, word‐part and phrase repetitions, and word breaks, with each feature tallied and normalized by utterance count. The reference domain included temporal‐reference errors, naming errors, paraphasias, hanging determiners, hanging topics, ambivalence, preposition omissions, incorrect determiners, definiteness repairs, and the number of mental‐verb uses, again quantified as error counts per utterance. For connectivity, we classified clauses as simple, coordinated, or subordinate and noted missing discourse markers; these data were used to calculate a structural complexity connectivity domain as ((1 × simple + 2 × coordinated + 3 × subordinate) / total clauses). Finally, the concordance domain recorded subject–auxiliary and subject–main‐verb person/number mismatches and inappropriate verb complements or forms, with error counts normalized by utterance count. A Spontaneous Language Composite Score (SLCS) was computed by averaging the normalized z-scores from the 4 linguistic domains. All transcripts were anonymized and scored by two independent investigators. Inter-rater reliability was excellent (Cohen’s κ > 0.90); any discrepancies were resolved by consensus.

**Supplementary Table 1 –** Domain scores of the Spontaneous Language Composite Score across groups

|  | *Healthy Controls N = 20* | *HD-ISS 0 N = 14* | *HD-ISS 1 N = 10* | *HD-ISS 2 N = 11* | *HD-ISS 3 N = 31* | *p-value* |
| --- | --- | --- | --- | --- | --- | --- |
| Fluency Domain | 0.07 (0.05, 0.11)c,d | 0.09 (0.06, 0.11)g | 0.10 (0.09, 0.13) | 0.14 (0.10, 0.15) | 0.11 (0.08, 0.18) | 0.016 |
| Reference Domain | 0.10 (0.08, 0.13)c,d | 0.07 (0.06, 0.09)f,g | 0.09 (0.05, 0.14)h,i | 0.15 (0.10, 0.18) | 0.16 (0.11, 0.30) | <0.001 |
| Connectivity Domain | 1.72 (1.63, 2.03)d | 2.00 (1.82, 2.27)f,g | 1.78 (1.70, 2.00) | 1.50 (1.30, 1.64) | 1.63 (1.20, 2.00) | 0.002 |
| Concordance Domain | 0.025 (0.017, 0.033)d | 0.022 (0.014, 0.028)g | 0.020 (0.014, 0.025)i | 0.022 (0.011, 0.043)j | 0.040 (0.030, 0.058) | <0.001 |

Data expressed as median (interquartile range). The Benjamini-Hochberg method was applied for multiple comparison correction, yielding adjusted p-values < 0.05.

aControls vs HD-ISS 0 *p <* 0.05

bControls vs HD-ISS 1 *p <* 0.05

cControls vs HD-ISS 2 *p <* 0.05

dControls vs HD-ISS 3 *p <* 0.05

eHD-ISS 0 vs HD-ISS 1 *p <* 0.05

fHD-ISS 0 vs HD-ISS 2 *p <* 0.05

gHD-ISS 0 vs HD-ISS 3 *p <* 0.05

hHD-ISS 1 vs HD-ISS 2 *p <* 0.05

iHD-ISS 1 vs HD-ISS 3 *p <* 0.05

jHD-ISS 2 vs HD-ISS 3 *p <* 0.05
